# Comment on Kokshoorn, B, and Blankers, BJ ‘Response to Grisedale, KS and van Daal, A: comparison of STR profiling from low template DNA extracts with and without the consensus profiling method’

**DOI:** 10.1186/2041-2223-4-2

**Published:** 2013-01-03

**Authors:** Kelly Grisedale, Angela van Daal

**Affiliations:** 1 Faculty of Health Sciences and Medicine, Bond University, Gold Coast, QLD 4229, Australia

**Keywords:** Low template DNA, Stochastic effects, Consensus profiling

## Abstract

Kokshoorn and Blankers responded to our recent article by saying that replicate analysis and consensus profiling of low template samples was best in terms of reliability and objectivity. We agree that the consensus approach has benefits, particularly in eliminating non-repeating spurious alleles from the final profile. However, with the development of statistical models that can accommodate stochastic effects and allele drop in, it may be beneficial to perform a single amplification with three times the amount of template, since much information is lost from the profile using the consensus approach.

## Background

In response to our recent article ‘Comparison of STR profiling from low template DNA extracts with and without the consensus profiling method’ [1], Kokshoorn and Blankers concluded that ‘one should distinguish between the quantity and the quality of information’, with quality being preferred when deciding on an analysis and interpretation strategy for low template DNA (LTDNA) profiles. This was in fact the conclusion of our paper. We agree that the consensus profiling works to reduce allele drop in (ADI) from the interpretation of the final consensus profile. However, other measures of quality, such as peak heights, peak height ratios and allele and locus drop out (ADO and LDO) were all improved when a single amplification with three times the amount of template was performed. We agree that consensus profiling has benefits, but it is important to note just how much information is lost from the profile when the consensus method is applied.

Kokshoorn and Blankers are correct in their response that our paper did not discuss the use of a stochastic threshold. Use of a stochastic threshold provides a means to confidently call alleles in mixture profiles. We believe that stochastic thresholds have value. However, with the increased sensitivity of LTDNA analyses, one must accept that most, if not all, results experience substantial stochastic effects and fall below a stochastic threshold. Our work, instead, was an exercise to simply observe how many alleles were obtained or lost when a consensus approach was implemented for single source samples. For that purpose we used the 50 RFU detection threshold implemented by others [2].

In casework, a stochastic threshold is an important component of LTDNA profile interpretation and the Netherland Forensic Institute’s use of a validated relative fluorescence units (RFU) threshold is to be lauded. However, Kokshoorn and Blankers’ statement that ‘a single analysis of all available template material in LTDNA samples, as proposed by Grisedale and Van Daal, will generally not yield a DNA profile of sufficient quality (that is, with all alleles of all donors above the stochastic threshold)’ and therefore any results from a single amplification are less objective than a consensus profile, seems odd, since there is no guarantee that a consensus profile will achieve this measure of validity either. Indeed, splitting a sample reduces the chance that any alleles in each of the aliquots can meet the stochastic threshold. Thus, under the Kokshoorn and Blankers approach most LTDNA samples would yield little information. In addition, if only alleles above the threshold are called in a consensus profile, then this approach can equally be applied to a profile from a single amplification.

Kokshoorn and Blankers are stating well accepted principles in their comments that mixture profiles involve more complex interpretations. This is true whether or not a consensus approach is applied. Alleles from a minor contributor should always be interpreted with care because of the potential for stochastic effects and the possibility of allele-sharing with a major contributor. Their points further support our position; allele-sharing and stochastic effects increase with decreasing amounts of total template DNA per amplification. However, statistical tools are being developed that may accommodate these issues and have been implemented in some laboratories.

In 2008, Gill *et al.*[3] stated, ‘There is currently no statistical model that incorporates all these parameters simultaneously. In this respect, all existing models must be considered incomplete (indeed we must consider that a complete model is unattainable)’. However, the statement following the aforementioned quote was, ‘However, the theory is well established, and the parameters of probability of dropout and probability of contamination can be universally applied to all DNA profiles using the framework originally described by Gill *et al.*[4], and programmed into an expert system [5,6]’. In the four years since this paper by Gill *et al.*[3] was published, further statistics software programs for the interpretation of mixture profiles have been developed which may accommodate ADO and ADI [7-10]. The consensus profiling method was initially developed to overcome the stochastic effects, particularly ADI [4]. If the statistical programs can incorporate these stochastic issues into the model, the evidence would be maximized by applying these tools to the DNA profile that contains the most information with the proper uncertainty/confidence associated.

Overall, our conclusions remain the same: the consensus approach will certainly be beneficial for dealing with ADI. However, when the template amount is limited, the most informative profile will likely be gained by a single amplification which can be analyzed using statistical interpretations that accommodate the stochastic effects and contamination.

## Abbreviations

ADI: Allele drop in; ADO: Allele drop out; LDO: Locus drop out; LTDNA: Low template DNA; RFU: Relative fluorescence unit. STR, Short tandem repeats.

## Competing interests

The authors declare that they have no competing interests.
